# Lifestyle Medicine Implementation in 8 Health Systems: Protocol for a Multiple Case Study Investigation

**DOI:** 10.2196/51562

**Published:** 2024-03-13

**Authors:** Meghan L Ames, Micaela C Karlsen, Samantha M Sundermeir, Neve Durrwachter, Tyler A Hemmingson, Melissa M Reznar, Kara Livingston Staffier, Bruce Weeks, Joel Gittelsohn

**Affiliations:** 1 Bloomberg School of Public Health, Johns Hopkins University Baltimore, MD United States; 2 American College of Lifestyle Medicine Chesterfield, MO United States; 3 School of Health Sciences, Oakland University Rochester, MI United States

**Keywords:** healthy lifestyle, implementation science, lifestyle medicine, multiple case study, noncommunicable diseases, prevention, qualitative methods

## Abstract

**Background:**

Lifestyle medicine (LM) is the use of therapeutic lifestyle changes (including a whole-food, plant-predominant eating pattern; regular physical activity; restorative sleep; stress management; avoidance of risky substances; and positive social connection) to prevent and treat chronic illness. Despite growing evidence, LM is still not widely implemented in health care settings. Potential challenges to LM implementation include lack of clinician training, staffing concerns, and misalignment of LM services with fee-for-service reimbursement, but the full range of factors facilitating or obstructing its implementation and long-term success are not yet understood. To learn important lessons for success and failure, it is crucial to understand the experiences of different LM programs.

**Objective:**

This study aims to describe in depth the protocol used to identify barriers and facilitators impacting the implementation of LM in health systems.

**Methods:**

The study team comprises team members at the American College of Lifestyle Medicine (ACLM), including staff and researchers with expertise in public health, LM, and qualitative research. We recruited health systems that were members of the ACLM Health Systems Council. From among 15 self-nominating health systems, we selected 7 to represent a diversity of geographic location, type, size, expertise, funding, patients, and LM services. Partway through the study, we recruited 1 additional contrasting health system to serve as a negative case. For each case, we conducted in-depth interviews, document reviews, site visits (limited due to the COVID-19 pandemic), and study team debriefs. Interviews lasted 45-90 minutes and followed a semistructured interview guide, loosely based on the Consolidated Framework for Implementation Research (CFIR) model. We are constructing detailed case narrative reports for each health system that are subsequently used in cross-case analyses to develop a contextually rich and detailed understanding of various predetermined and emergent topics. Cross-case analyses will draw on a variety of methodologies, including in-depth case familiarization, inductive or deductive coding, and thematic analysis, to identify cross-cutting themes.

**Results:**

The study team has completed data collection for all 8 participating health systems, including 68 interviews and 1 site visit. We are currently drafting descriptive case narratives, which will be disseminated to participating health systems for member checking and shared broadly as applied vignettes. We are also conducting cross-case analyses to identify critical facilitators and barriers, explore clinician training strategies to facilitate LM implementation, and develop an explanatory model connecting practitioner adoption of LM and experiences of burnout.

**Conclusions:**

This protocol paper offers real-world insights into research methods and practices to identify barriers and facilitators to the implementation of LM in health systems. Findings can advise LM implementation across various health system contexts. Methodological limitations and lessons learned can guide the execution of other studies with similar methodologies.

**International Registered Report Identifier (IRRID):**

DERR1-10.2196/51562

## Introduction

Cardiovascular disease, diabetes, and other chronic illnesses (including mood disorders) continue to be the leading causes of morbidity and mortality in the United States [1,2]. An estimated US $4.1 trillion is spent annually on health care for individuals with chronic physical and mental health conditions [2]. Poor health behaviors, such as a less healthy diet, physical inactivity, and substance use, exacerbate these conditions and cost the United States an estimated US $1.3 trillion per year [3]. An estimated 75%-90% of chronic illnesses could be prevented by lifestyle modification [4].

Lifestyle medicine (LM) is a “medical specialty that uses therapeutic lifestyle interventions as a primary modality” to prevent, treat, and reverse chronic conditions [5]. This “evidence-based, whole-person, prescriptive lifestyle change” is built around 6 pillars: a whole-food, plant-predominant eating pattern; regular physical activity; restorative sleep; stress management; avoidance of risky substances; and positive social connection [5]. These innovative and comprehensive approaches, especially those related to diet, have been shown to promote healthy weight, decrease the risk of type 2 diabetes, reduce cardiovascular risk factors, and improve quality of life [6-15]. Experts estimate US $116 billion could be saved annually through modest changes in health behavior and care delivery that result in improved treatment rates, increased physical activity, reduced smoking, and reduced obesity [16].

Interest in LM is growing, as evidenced by the rising membership of the American College of Lifestyle Medicine (ACLM), which increased by approximately 300% between 2018 and 2021 [17]. In 2017, the American Medical Association House of Delegates emphasized the importance of LM treatment by passing a resolution supporting providers to prescribe healthy lifestyle behaviors [18]. Despite these advances, adoption in clinical practice is slow [12,13,19-21], and additional research is needed to better understand the barriers and facilitators to implement this approach in health care settings [20].

The Consolidated Framework for Implementation Research (CFIR) is commonly referenced in the implementation science field to characterize factors that can influence the successful implementation of health services [22]. It includes 5 domains: intervention, inner setting, outer setting, individuals involved, and the implementation process. About 13% of the estimated 625 health systems across the United States are members of the ACLM Health Systems Council and are at varying stages of LM implementation [23,24]. Commonly, the inner setting of LM takes place within primary care and the general internal medicine environment, as well as medical specialties such as diabetes and oncology. Implementation processes and strategies can often extend across disciplines, such as the case with the modification of electronic medical records to prompt behavioral screening and referrals. Systematic implementation of LM faces various challenges, including inadequate leadership support and clinician training in the inner setting and patient preferences and reimbursement complexities in the outer setting [19,20,22,25]. A 2019 survey of ACLM members assessed respondents’ current practice of LM, including reimbursement, quality measures, and patient outcomes [26]. In reviewing findings (published elsewhere), ACLM leadership and consultants determined that greater critical richness and additional details could be achieved through a qualitative examination of a subset of US health care systems with LM programs.

ACLM’s mission calls for additional high-quality research, education, and advocacy to continue building evidence that LM should be more comprehensively implemented by US health care systems [27]. However, the current literature lacks documentation of the most effective implementation strategies to support LM practice. To address this gap, we are conducting a multi–health system case study to understand the factors that led to the development, growth, and maintenance of successful LM programs within health systems. Specific aims of *Lifestyle Medicine Integration in Health Systems: A Case Study Project* include: (1) examining in-depth 8 LM programs and constructing a detailed case narrative report for each system; (2) identifying factors influencing the initiation and growth of LM practices; and (3) describing common facilitators and barriers across health systems to the continued implementation of LM.

This paper outlines the study protocol, including case selection, data analysis, and dissemination of research findings. It will aid in interpreting study findings and advise the research execution of other studies that incorporate similar methodologies across various settings.

## Methods

### Overview

In response to a gap in the rich understanding of barriers and facilitators to implementing LM in health systems, the research team selected a qualitative research approach investigating multiple health systems as individual case studies. This multiple case study approach incorporates insights from multiple instances of the phenomenon of interest [28,29].

This paper describes the 8 steps used to conduct this research, as outlined in Table 1. At the time of this writing, steps 1-5 have been completed and steps 6-8 are in progress.

**Table 1:** Protocol steps for the Lifestyle Medicine Integration in Health Systems study.

Step	Activity	Status^a^
1	Formation of the study team	Complete
2	Selection of methods	Complete
3	Recruitment and training of the study team	Complete
4	Case study nomination and selection	Complete
5	Iterative data collection	Complete
6	Iterative preparation of case study narratives	In progress
7	Preparation of cross-case reports	In progress
8	Dissemination of findings	In progress

^a^As of February 1, 2024.

### Step 1: Formation of Study Team (Completed)

The study team comprises team members at ACLM staff and researchers with expertise in public health, LM (including behavior change), and qualitative research. As the national medical professional organization representing physicians and other health professionals practicing LM, ACLM is uniquely positioned to form and coordinate this study team. In addition to representing individual practitioners, ACLM also coordinates a network of health systems, the Health Systems Council (HSC). This group comprises integrated health systems around the United States committed to growing their LM service offerings and sharing their experiences and learnings with other council members [24]. In July 2021, the study team initiated regular (weekly or biweekly) meetings to discuss research aims and methods. The study team has continued to meet throughout the duration of the project.

### Step 2: Selection of Methods (Completed)

Through discussion at regular meetings and review of qualitative literature, the study team identified the methods that would be used for this project. We selected a qualitative case study approach, embracing constructivist epistemology (the belief that there is no one truth and that findings are created by the interaction between the patients or informants and the observer or data collector) [30]. Case studies are a qualitative methodology that facilitates the exploration of a specific topic through the development of complex narratives, promoting rich insights from multiple sources within single and multiple cases [28,31,32]. Although findings are heavily influenced by contextual factors, the cross-case reports (described later) provide insights that can be transferred to other settings. This need for transferability suggests a multiple case (rather than single-case) design would be appropriate for the identified research questions [32].

We selected case studies to allow for contextual diversity and triangulation, or the gathering of evidence from multiple data sources, to yield convergent findings [28,33]. To facilitate triangulation, the study methodologies included data collection from multiple stakeholders and source types (in-depth interviews, document review, survey responses, and direct observation). Further, document review allows for the gathering of evidence by interviewers ahead of interviews and reduces the burden on informants [32]. We selected in-depth interviews instead of focus groups to allow for triangulation from different perspectives (both individual experiences and professional roles) and reduce social desirability bias and fear of disclosure among informants [34]. Finally, direct observation (in the form of in-person site visits) yields insights that interviewees may have accidentally omitted due to their own familiarity with their particular context.

### Step 3: Recruitment and Training of Study Team (Completed)

The study team is coled by the principal investigator (MCK) and a senior investigator with expertise in qualitative research and case study methods (JG). Additional expert advisors representing ACLM (TAH and KLS) and academic institutions (MLA, SMS, ND, and MMR) offer critical insights and guidance to protocol development and implementation. All data collection was completed by researchers who are external to ACLM, apart from 2 students who were previously involved as members of ACLM. ACLM staff advise on study design or implementation, participate in team meetings, and are part of the iterative review process, but do not conduct data collection.

There were 8 data collectors, who primarily comprised graduate-level students trained in the fields of public health and medicine. Data collectors were hired as research assistants and recruited through study team networks. All data collectors completed data collection training designed and delivered by the academic consultant. The training included general principles of qualitative research, qualitative interviewing techniques, and a review of the study protocol.

### Step 4: Case Study Nomination and Selection (Completed)

Multiple case studies are recommended to contain 4-10 cases to achieve sufficient variability while providing a manageable amount of transferrable insights [28]. Stake [28] identifies 3 criteria that should be present when selecting cases: (1) the case must be relevant to the quintain (or phenomenon of interest, which is LM implementation); (2) the cases must provide contextual diversity; and (3) the cases must allow researchers to observe and explore complexities and contexts.

We followed a purposive sampling approach that leveraged the preexisting ACLM HSC network, which is uniquely positioned to access health systems with LM programs. From March 21-April 1, 2022, recruitment emails were disseminated through ACLM HSC email communications. During the recruitment period, 15 health systems were self-nominated by an employee representative from each interested health system. The self-nomination form captured data on the health system's geographic location, patient demographics, payer types, LM practitioners, programs available, and the estimated reach of the LM program.

Table 2 includes selected self-reported data for the participating health systems and describes geographic location by census-based US regions to preserve health system anonymity [35]. Data reported through the nomination form were not corroborated and should be interpreted conservatively. One health system (site code H) did not complete the nomination form and was recruited through a different mechanism, described below.

**Table 2:** Summary of self-reported characteristics of participating health systems.

Site code	Region	Level of focus^a^	Reach^b^
A	South	Subspecialty	Small
B	West	Specialty	Large
C	Midwest	Subspecialty	Medium
D	West	Specialty	Medium
E	South	Specialty	Medium
F	Midwest	Subspecialty	Small
G	West	Specialty	Large
H	South	—^c^	—

^a^“Specialty” indicates lifestyle medicine is a stand-alone area of treatment programming; “subspecialty” indicates lifestyle medicine is an adjunct approach embedded in other treatment specialties.

^b^Reported estimated number of patients receiving care at the time of nomination, where “large” is >5000, “medium” is 1000-5000, and “small” is <1000.

^c^Not available.

Study team members reviewed all self-nomination forms and came to a consensus through discussion about which cases to include to achieve contextual diversity, including variability in program size, age, geographic location, payer model, and population served. We selected 4 instrumental cases that were generally representative of the “typical” nominees seen but varied in the aforementioned characteristics [28]. We also selected 2 intrinsic cases that offered a unique context due to their stage (either very early or relatively mature in development), size, and extent of LM practice integration [28]. Selected cases were invited to confirm participation through a health system representative authority.

Partway through case recruitment and data collection, the study team determined that a contrasting case was needed to demonstrate the experiences of a health system that had initiated and then aborted an LM program. The study team agreed that this perspective would yield unique insights about implementation barriers to LM. This iterative approach is an accepted multiple case study procedure, through which redesign can emerge partway through case selection [32]. Original recruitment strategies did not satisfy this need, and the study team used an additional recruitment strategy that built upon individual communications rather than wide-reaching HSC communications channels. One case (site code H) was recruited using this approach and varied slightly in the data collection methods described below.

### Step 5: Iterative Data Collection (Completed)

#### Overview

Each participating health system was assigned a single data collector as a site lead, whose responsibilities included coordinating data collection and drafting the case narrative. The site lead managed a team of 1-2 other data collectors, who conducted individual interviews with different members of the health system’s LM team. Overall, 4 types of data were collected: in-depth interviews, site visits, existing documents, and study team notes.

#### In-Depth Interviews

The study team conducted at least 6-8 in-depth interviews with individuals identified in each participating health system. Health system liaisons were asked to identify employees who were integral to the implementation of the LM programming. They were provided a list of potential types of roles sought for interviews and asked to prioritize individuals who served as health system leaders or administrators (including billing professionals) and physicians. Other health care professionals delivering LM were also requested and included nurse practitioners, registered dietitians, behavioral health specialists, health coaches, exercise physiologists, physical therapists, kinesiologists, and mental health professionals. Only in the instance of the contrasting case were former employees also invited to participate in interviews.

Interviews lasted 45-90 minutes and were conducted through video call (typically), telephone (rarely), or in-person (rarely). When using video call, interviewees had the option of participating with their camera on (typically) or off (rarely). Interviews were open-ended and exploratory and followed a semistructured interview guide designed to achieve the study aims and allow for adaptability based on the interviewee or health system context. The interview guide was iteratively designed by study team members and loosely structured on the CFIR model [22]. Minor updates to the interview guide were made as emergent topics were identified.

In alignment with the CFIR, interviewees were asked about the inner (structural characteristics, culture, and available resources) and outer (patient population, billing, or payer practices) settings of their health system in addition to individual factors (their specific role and the roles of others) [22]. They were also asked to describe their understanding of the intervention (LM), how it differs from other types of medicine within their setting, and how it was adapted for their specific setting. The interview also included questions about barriers, facilitators, and processes related to program launch and growth. Interviewers were trained to probe topics particularly relevant to the interviewee or health system context. If warranted, the interviewer requested a follow-up interview with an interviewee. These follow-up interviews were intended to answer specific questions, provide missing details, or explore a topic not previously discussed. The most recent version of the interview guide at the time of publication is available in Multimedia Appendix 1.

The 2 study team members assigned to each participating health system were responsible for participating in each associated interview, either as a primary interviewer or as an interview reviewer, which involves reviewing the transcript. Interviews were recorded using the video meeting software (Zoom; Zoom Video Communications [36]) or a recording device (or sometimes both for redundancy) and stored in a secure location on the cloud. Recordings were transcribed using the Microsoft One Drive (Microsoft Corporation) transcription and then manually reviewed and edited for accuracy by study team members. Additionally, interviewers documented emerging themes or other contextual factors following each interview.

#### Document Review

Interviews were supplemented by the review of available health system documentation. The study team collected and reviewed publicly available annual reports, websites, program promotional materials, strategic plans, and relevant community health needs assessments. Interviewees were asked about additional materials they thought were relevant to the interview themes, and such materials (which may include organizational charts, internal planning documents, and patient education materials) were also reviewed. The study team maintained a database of all reviewed documents to aid in identifying additional documents for review. Interviewers referenced documents if and as needed when reflecting on and taking notes about past interviews, preparing for upcoming interviews, and sharing updates during study team discussions. For example, if an interviewee mentions an LM program that is referenced by multiple names, the interviewer can reference the health system website and patient recruitment materials to confirm the official name of the program. Additionally, documents are referenced during the preparation of the case study narratives and cross-case analysis to corroborate findings, fill in missing details, and provide illustrative examples. For example, if a health system reported promoting referrals by sending email communications to physicians, the language from the communication may be included in the case study narrative as an example of that recruitment strategy.

#### Site Visits

An in-person site visit occurred for 1 site and included unstructured observations of the settings and conversations with health system employees, which mirrored themes included in the in-depth interview guide. Photos from the site visit (including patient waiting areas, exam rooms, dining and exercise facilities, offices, and other available areas) are referenced in the qualitative data analysis. Otherwise, site visits were not conducted. This decision was made due to the restrictions of the COVID-19 pandemic.

#### Weekly Study Team Discussions and Iterative Review (Currently in Progress)

Following the principle of emergent design [37], the study team meets 2-4 times per month to debrief from interviews and discuss emerging themes. During team meetings, data collectors report on themes from recent interviews, and the study team discusses how findings enhance the understanding of health system LM implementation and identifies areas to probe further in future interviews. This is also when the study team discusses similarities and differences among health systems and determines the need for potential changes to the study protocol, case selection, interview guide, case narrative outline, and cross-case analysis. Additionally, study team members report on current events and publications that are relevant to research aims. Meeting recordings and minutes are available for study team members to reference during data analysis.

### Step 6: Preparation of Case Study Narratives (Currently in Progress)

In collective case study analysis, it is prudent to conduct individual analysis initially (described below) and follow with cross-case analysis (described in step 7) [38]. Through an iterative and collaborative process, the study team developed a case report format that is followed for the preparation of each case narrative to facilitate intercase comparison. This requires that case narratives open with a presentation of objective data, including name, location, size, payer model, etc. The following sections align with the overall study aims and include potential barriers and facilitators to initiation and sustainment. After completing 2 of the case study narratives, the study team decided to add 2 additional sections focused on clinician training and provider burnout to capture emergent themes. All case reports will follow the same report template (the original 2 case study narratives were revised to align with the updated structure) but vary in length and subtopics covered specific to each health system. The case narrative template is included in Multimedia Appendix 2.

The site’s lead data collector is primarily responsible for writing the case study narrative. Other study team members review and comment on case narrative drafts until a consensus is reached. As appropriate and necessary, study team members may conduct additional follow-up interviews or share versions of the case narratives with interviewees (with individual identifiers removed) to facilitate member checking. Additionally, 1 team member is responsible for reviewing all reports for consistency in form, content, and style.

### Step 7: Cross-Case Analysis (Currently in Progress)

Following the completion of case study narratives, the study team completes cross-case analyses focused on topics of interest. Specific analysis topics are not yet final but will likely include billing, care delivery models, clinician training, leadership support, buy-in, intervention content, workplace culture, and burnout. Each cross-case analysis will yield a manuscript to be submitted for peer-reviewed publication. Because the cross-case analyses are not yet complete, the following methods offer a high-level overview of the procedures planned for each analysis report.

Some cross-case analyses will follow the multiple case study methodology reported by Stake [28], which includes the following steps: (1) plan the cross-case analysis and identify themes relevant to research questions; (2) become familiar with individual cases; (3) assess case utility for each cross-case theme; and (4) sort and merge findings relevant to themes. In the Stake [28] approach, a series of interactive worksheets are completed to identify emergent themes, guide analysts through their review of case narratives, and plan and execute a cross-case report. This case study analysis methodology was selected as it preserves contextual information to a large degree. Other cross-case analyses may leverage a coding approach, which uses search queries to identify relevant segments of transcripts or documents and then applies an inductive coding schema to the resulting data.

### Step 8: Dissemination of Findings (Currently in Progress)

The study team plans to disseminate a series of manuscripts. This paper offers an overview of the methods conducted and planned for data collection, analysis, and reporting. We also plan to disseminate shortened, deidentified versions of the case study narratives to serve as vignettes for consideration by other practitioners. Additional papers will provide insights for each of the cross-case analyses by comparing and contrasting specific findings for each case study site and highlighting common practices seen in instrumental cases and unusual situations seen in intrinsic cases [28]. These can advise on the implementation and integration of LM programs into health systems that can be applied in other settings to initiate or scale current LM offerings.

Additionally, we plan to share deidentified case narratives with the respective participating health systems. This serves the dual purpose of (1) facilitating member checking and (2) offering the benefit of an external perspective to participating health systems.

### Ethical Considerations

The University of New England’s institutional review board (IRB) reviewed the study protocol and determined it was exempt from IRB review and oversight (project number 1221-21). Before the interview, all participating individuals completed a written informed consent, which included an overview of the study purpose, a request for participation, a description of privacy protection efforts, and a review of potential risks and benefits. Participants were not compensated for their time.

## Results

At the time of this writing (February 1, 2024), the study team has completed all the interviews at 8 health systems. The team interviewed 63 individuals, 5 of whom participated in a follow-up interview, resulting in 68 total interviews. Every site included an interview with at least 1 administrator and physician. Across all sites, interviews were conducted with 25 health system leaders or administrators; 16 physicians; 7 registered dietitians; 6 behavioral health specialists or health coaches; 4 nurse practitioners; 2 exercise physiologists, physical therapists, or kinesiologists; 2 mental health professionals; and 1 individual with an unclassified role.

The study team has completed initial drafts of all 8 case study narratives and is abridging and deidentifying them for member checking and broader dissemination. Cross-case analysis is underway to identify critical facilitators and barriers, explore clinician training strategies to facilitate LM implementation and develop an explanatory model connecting practitioner adoption of LM and experiences of burnout. Additional analyses may investigate how billing strategies, care delivery models, leadership support or buy-in, and intervention content can impact LM implementation in health systems.

## Discussion

### Overview

This research is the first multiple case study examining facilitators and barriers to LM implementation in health system settings. It will address a gap in the literature by providing insight into the barriers and facilitators to adopting LM practice in health systems. Below is a discussion of limitations, strengths, and opportunities for further research.

### Limitations

ACLM serves as the primary funder for this work, potentially introducing a pro-LM bias. To reduce the influence of this bias, the study team was intentionally designed to be diverse and include experts who are external to ACLM and thus not biased in the same way that ACLM affiliates are. Additionally, all methodological and analysis decisions are made during study team meetings, which include multiple perspectives, including 1 senior advisor, in addition to ACLM.

In some cases, the study team was challenged to identify interviewees who comprehensively represented the breadth of LM activities in a single health system. This is partly due to the size and complexity of participating health systems. Individual interviewees were identified by 1 or 2 liaisons at each health system and sometimes did not include groups or types of individuals who would have offered valuable insights. For example, 1 site provided names of individuals who were involved in implementing one specific LM program but were not familiar with the LM residency program that was offered by a different unit of the health system. This resulted in in-depth data about the specific LM program but fewer insights into the LM residency offerings. Additionally, some health systems were unable to allow nonexempt employees to participate in in-depth interviews because the study was not compensating interviewees for their participation. This resulted in an overrepresentation of leadership and an underrepresentation of front-line workers and intervention delivery personnel. To address these challenges, the study team circled back to health system liaisons to request interviews with additional individuals whose perspectives were not initially included, although the requests were not always met. Data collection methods did not directly include patients or community members, resulting in a gap in these perspectives. Interviewees were asked to speak about the experiences of these individuals, but future research should also investigate these perspectives, specifically.

Finally, site visits and in-person interviews were limited due to restrictions associated with the COVID-19 pandemic. We were only able to conduct 1 site visit, which was conducted in person. To adapt, we revised our methods to prioritize internet-based interviews. Through this process, we learned that internet-based interviews facilitated easier scheduling and access to individuals and often yielded high-quality interviews that were thought to be comparable to in-person interviews. Considering the added logistical benefits, the study team determined the internet-based interview approach to be preferable to in-person site visits. No interviews were conducted in person.

### Strengths

This multiple case study methodology and our iterative team-based approach are strengths of this research. By preserving the context of each case, researchers can gain a rich understanding of the many factors impacting the phenomenon of interest [28,31]. Although the selection of ACLM HSC members allows for insights into the experiences of early adopters, the findings may not be translatable to relatively more nascent programs. Throughout the early stages of case selection, our team reviewed health system components to ensure variability in program maturation. We intentionally selected health systems with more recently established LM programs and also sought out and recruited a contrasting case that had initiated and then greatly reduced LM programming. However, the study would have been further strengthened by the inclusion of additional negative cases. The heterogeneity of participating health systems will contribute to the transportability of the study findings. Future research should investigate if and how barriers and facilitators are different among health systems that are not currently aligned with LM.

A strength of this study is that the cases and analyses take place at the health system level, allowing a more comprehensive perspective that incorporates all CFIR domains [22]. Health systems are complex systems of hospitals, clinics, and individuals connected through joint ownership or management [38]. Within these systems are varying cultures (“individuals involved” domain), policies, and processes (“inner setting” domain), which can impact practices (“process domain”). Examination at the health system organization level offers insights into the macrolevel factors (“inner” and “outer setting” domains) that impact LM implementation. Gaining permission to work with such large and complex organizations can be challenging, however, and in some instances, investigation was delayed and even prohibited due to the inability or unwillingness of health system leadership to provide permission for participation. Some health system leaders expressed confidentiality concerns, noting that patient perspectives of the organization and their practices were a critical consideration.

### Conclusions

This protocol paper offers real-world examples of research methodologies used to gather data on a series of health systems. Additionally, the study findings will yield practical insights into strategies to effectively implement LM in health systems. Health system leaders and administrators can draw on these findings to establish and grow their own LM programs and integrate LM practices into existing services. Expanded access to LM treatment may result in improved morbidity and mortality outcomes related to chronic diseases [1,6,12].

## Appendix

Multimedia Appendix 1 In-depth interview guide.

Multimedia Appendix 2 Case study narrative outline.

## Abbreviations

ACLM: American College of Lifestyle Medicine
CFIR: Consolidated Framework For Implementation Research
HSC: Health Systems Council
IRB: institutional review board
LM: lifestyle medicine

## Data Availability

The data presented in this study cannot be shared due to privacy concerns and the inability to adequately secure the identity of the participants.
