# Supplementary material for: Lifestyle Medicine Implementation in 8 Health Systems: Protocol for a Multiple Case Study Investigation
Source: JMIR Res Protoc. 2024 Mar 13;13:e51562. doi: 10.2196/51562 (PMC10973966; doi:10.2196/51562)
Supplement: Multimedia Appendix 1 [file resprot_v13i1e51562_app1.docx]

**Lifestyle Medicine Case Study**

**In-Depth Interview Guide**

Introduction

- Thank you for meeting with me today. I’m interested in learning about how lifestyle medicine practices are implemented in your health system.
- Interviewer reviews with the participant the contents of the Informed Consent Form signed and allows them an opportunity ask any questions or express concerns.
- The questions I am going to ask don’t have right or wrong answers. Remember that this conversation is completely confidential and we can skip any question you prefer not to answer.
- Is it okay if I take some notes?
- Capture name and title
- **Note for interviewer:  The questions listed after each bullet point are sample probes.  It is not expected that you will ask every question listed.**

Questions

- 1. Tell me about your practice/the work you do and in what way your work includes LM.

1. Probe on their specific position/roles
2. Probe on work in LM

1. From your perspective, what is LM? How is LM differentiated from other types of medicine in your system?

1. Tell me about how you have incorporated LM into your practice/the work you do.
2. Walk me through the program(s) currently in use, including the timeline, areas of LM covered, and behavioral strategies used.
3. Probe to paint a picture of specific programs
4. Probe on behavioral strategies
5. Probe on environmental changes
6. Etc.
7. Who is involved in implementing the program and what are their roles?
8. What factors led to LM to gain traction in your system? We’re interested in whether there were different stages of growth or specific events that contributed to or hindered the development?
9. Probe to gain further insight based on answers
10. Has your offering of LM programming had any kind of effect on your ability to attract new patients and capture more market share?
11. What kind of resources are earmarked for the LM practice/program in your system? (e.g. money, staff, space, etc.)
12. How does the patient population the LM practice/program serves differ from your overall patient population?
13. Overall patient population
14. LM patient population
15. Are there criteria for patients seen/included?
16. What kind of feedback do patients give you?

1. How do you incorporate the [nutrition] domain of LM into your practice?
2. Ask for each domain (nutrition, physical activity, stress, sleep, relationships/social support, and substance use).

1. Can you talk about the administrative support, or challenges, related to administrative support for implementing LM in your health system?
2. Examples: Staffing, billing, program design, scaling, familiarity, etc.

1. What environmental or cultural factors would enhance your LM offerings?
2. Building resources? (teaching kitchen, PA area? Etc.?)
3. Cultural competency in staff?
4. Insurance issues?

1. What resources or other changes would enhance your LM offerings?
2. Probe on specific information, training, materials needed.
3. What types of training activities does your health system offer or do health system team members participate in?
   1. What strategies are utilized to train clinicians?
      1. Consider mentorship, continuing education, grand rounds, in-services, residency programs, certification programs, conferences, etc.
   2. Who are key partnerships leveraged for clinician training?
      - 1. Who developed the training programs? Was it clinician employees? Leadership? External partners?
        2. Has the health system partnered with any groups/organizations to training clinicians?
        3. How (if at all) has the health system partnered with ACLM?
   3. How are training activities funded?
4. Can you talk about your experience with burnout?
5. What lessons have you learned that you would like to share with other health systems who are interested in offering LM care?

1. Who else should I speak with to get more information on (design, content, pillar area, program, how used, etc.)?
2. I am interested in reviewing any written/electronic materials you could share with me regarding (the persons work on LM in the health system). This would include reports, websites, pamphlets, internal/external release materials, curricula, press coverages -- I do not want any sort of protected or confidential information.
   - 1. How could I go about accessing these materials?
     2. Who would I need to contact to access these materials?
