# Supplementary material for: Lifestyle Medicine Implementation in 8 Health Systems: Protocol for a Multiple Case Study Investigation
Source: JMIR Res Protoc. 2024 Mar 13;13:e51562. doi: 10.2196/51562 (PMC10973966; doi:10.2196/51562)
Supplement: Multimedia Appendix 2 [file resprot_v13i1e51562_app2.docx]

**Lifestyle Medicine Case Study Report Outline**

**Overview:**

This case report outline serves as a guide to writing the case reports for each site. We expect all case reports to include the main section headers (labeled with numbers) in the order in which they appear, but you can feel free to use your own subheadings, and organize each main section as appropriate for your site. Because each site is different, this outline is meant to allow for flexibility within each section to include the most important themes, and leave out anything that is irrelevant depending on the site.

**General Format:**

1. 12 pt font, single spaced
2. Italicized relevant quotations embedded throughout (indented if >1 sentence)
   1. Identifier at the end (e.g. idi #13)
3. Integration of data sources (qualitative and quantitative) within each section (also including lay press articles, photos)
4. Use of tables/figures in common formats
   1. Some sections could be presented in a more visual format (e.g. timeline)

**Report Outline:**

**Title: Health System X: An Established LM program**

**At the top: Basic stats on the health system: e.g., population served, size of population, etc. (just a few numbers to allow the reader to sort or classify the case studies). Consider including LM pillar scores here**

1. Executive Summary (~ 1 page)
   1. Write this section last
   2. Include key lessons learned from the case
   3. Include recommendations (this may overlap with lessons and be unneccesary)
2. Methods (Separate text box)
   - 1. Table of each position/role type and number of interviews
     2. Written, electronic documents, websites, etc.
     3. Visits done, observation, etc.
     4. The protocol has more detail about data collection, training, analysis – reference that paper here instead of repeating in each case report.
     5. Describe any limitations/restrictions (intentional or unintentional) in the representation of the interviews
3. Introduction (~ 2 pages)
   1. Site Setting
      1. Type of health system
      2. Location
      3. Population served
         1. Payer types
         2. Income, race/ethnicity
         3. Chronic conditions
      4. In table format: Size, number of patients served
   2. Operational details
      1. In table format: Organizational structure (admin, staff, types of clinicians, etc.)
4. Lifestyle Medicine Timeline (2+ pages)
   1. Open with a high-level timeline (figure)
   2. Narrative description of different phases
      1. Program initiation—Factors/events that led to the start of LM programs (may be in timeline form)
         1. Discuss events, people, funding, policies, leadership that were critical at this phase
      2. Program growth—Factors/events that helped LM programs gain traction and growth (may be in timeline form), such as time, resources, funding, policies, leadership, etc.
         1. discuss events, people, funding, policies, leadership that were critical at this phase
      3. Program sustainability
         1. discuss events, people, funding, policies, leadership that were critical at this phase
5. Lifestyle Medicine Programs Offered (2+ pages)
   1. Describe this within Patient, Community, and Employee realms as applicable (include these as major sub-headers)
      1. Description of formal programs offered (Venn diagram)
         1. Name of the program (CHIP, DPP, etc.) or description if there is not a structured program
            1. Topic areas of LM covered (do you mean pillars?)
            2. Number of sessions, length of each session, if applicable
            3. Behavioral strategies used, environmental changes encouraged, etc.
            4. Number of patients who have completed the program

Patient outcomes if reported in the written documents that we have. For example, MedStar provided a report that includes general stats on patient satisfaction with the program that could be included here. [no actual patient data]

- - 1. Coverage of the LM Pillars (this could be a table, text or both – a matrix with “scores” would make the different pillars and cases studies comparable)
       1. Nutrition
       2. Physical activity
       3. Stress
       4. Sleep
       5. Substance Use
       6. Social support

1. Key People Involved in Program Implementation Today (~2 pages)
   - 1. Staffing models
     2. Types of expertise available (e.g., LM-licensed clinicians, dietitians, exercise physiologists, etc.)
     3. Types of patients who participate
        1. eligibility criteria
2. Marketing Practices (~2 pages)
   1. How LM players market the program to other sites/clinicians
   2. How LM players market the program to patients
      1. How are they recruited?
3. Barriers and Facilitators to Initiating and Sustaining Lifestyle Medicine Programs at [insert site name] (2+ pages)
   1. This includes, but is not limited to:
      1. Administrative support
      2. Site-specific cultural factors that enhance or detract from LM
      3. Staffing
      4. Billing
      5. program design
      6. scaling
      7. resources needed/changes needed to enhance LM offerings
4. Future Plans for Expanding/Improving the LM programs
5. Lessons Learned (~1 page)
   1. Combine with IDI data as well as case report writer insights
      1. What advice did interviewees give related to starting and sustaining LM programs?
6. Appendices
   1. Any written materials we are allowed to include here
   2. Site photos taken
7. Amendments
8. LM Training
   1. Penetration of LM certification and LM knowledge
   2. Barriers and facilitators identified to translating knowledge. Consider timing, staffing burden, interest, leadership
   3. Strategies utilized to train clinicians. Consider mentorship, continuing education, grand rounds, in-services, residency programs, certification programs, conferences, etc.
      1. What types of enhancement activities does the health system offer? Consider consultation, self-reflection, incentivizes, dedicated time for training, supported learning, community of practice groups.
   4. Key partnerships leveraged for clinician training
   5. Funding mechanisms (consider employer reimbursement, scholarships, health system budgets, etc.)
   6. What are the health system goals for expanding training?
